# Timing is everything: cell cycle control of Rad52

**DOI:** 10.1186/1747-1028-5-7

**Published:** 2010-02-23

**Authors:** Jacqueline H Barlow, Rodney Rothstein

**Affiliations:** 1 Experimental Immunology Branch, National Cancer Institute, National Institutes of Health, Building 10, Room 4B04, 10 Center Drive, Bethesda, MD 20892, USA; 2 Department of Genetics & Development, Columbia University Medical Center, 701 West 168th Street, HHSC 1608, New York, NY 10032-2704, USA

## Abstract

Regulation of the repair of DNA double-strand breaks by homologous recombination is extremely important for both cell viability and the maintenance of genomic integrity. Modulation of double-strand break repair in the yeast *Saccharomyces cerevisiae *involves controlling the recruitment of one of the central recombination proteins, Rad52, to sites of DNA lesions. The Rad52 protein, which plays a role in strand exchange and the annealing of single strand DNA, is positively regulated upon entry into S phase, repressed during the intra-S phase checkpoint, and undergoes posttranslational modification events such as phosphorylation and sumoylation. These processes all contribute to the timing of Rad52 recruitment, its stability and function. Here, we summarize the regulatory events affecting the Rad52 protein and discuss how this regulation impacts DNA repair and cell survival.

## Introduction to double-strand break repair

**Figure 1:**
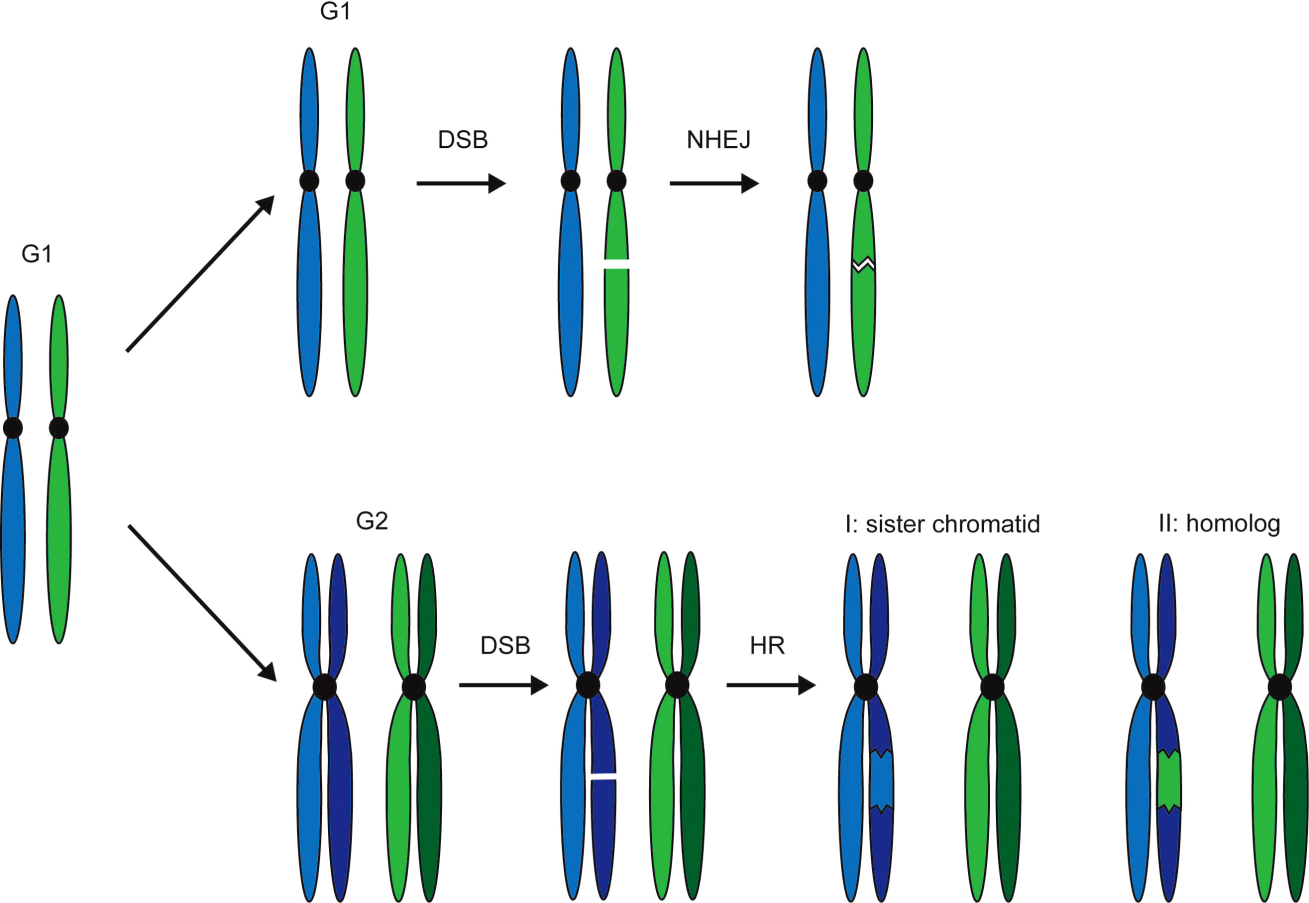
**Repair of a DSB proceeds according to cell cycle stage**. In G1, cells have a single copy of each chromosome (light blue and light green). If a break occurs in G1, the cell repairs the DSB by NHEJ, directly resealing the DNA ends (top). In G2, after chromosomes have been replicated, there is a sister chromatid, an identical copy of each original chromosome (dark blue and dark green, bottom). When a DSB occurs in G2, it is normally repaired by HR using either the sister chromatid or a homologous chromosome as a template. Repair from the sister (I) results in restoration of the exact information lost at the break site. Repair from the homolog (II), however, may lead to loss of heterozygosity if accompanied by a crossover.

Double-strand breaks (DSBs) can be repaired by two major pathways, non-homologous end-joining (NHEJ) or homologous recombination (HR). NHEJ directly rejoins broken DNA ends by ligation [1,2], while HR utilizes a homologous DNA template to prime DNA synthesis and restores genetic information lost at the break site (Figure 1; [3-5]). Both NHEJ and HR follow stepwise pathways leading to repair that involve distinct sets of proteins. In both pathways, the MRX complex--comprised of Mre11, Rad50 and Xrs2--first recognizes and binds the exposed ends of the DSB [6-10]. In NHEJ, the yeast Ku70/Ku80 heterodimer also recognizes and binds the DNA ends. Ku70/Ku80 then recruits the Lif1/Nej1 heterodimer and Lif1 both recruits and stimulates Dnl4 ligase activity to complete repair [1,11-14]. Processing of the ends for NHEJ is limited, and NHEJ itself is potentially mutagenic, resulting from the loss of genetic material at the break/join site.

**Figure 2:**
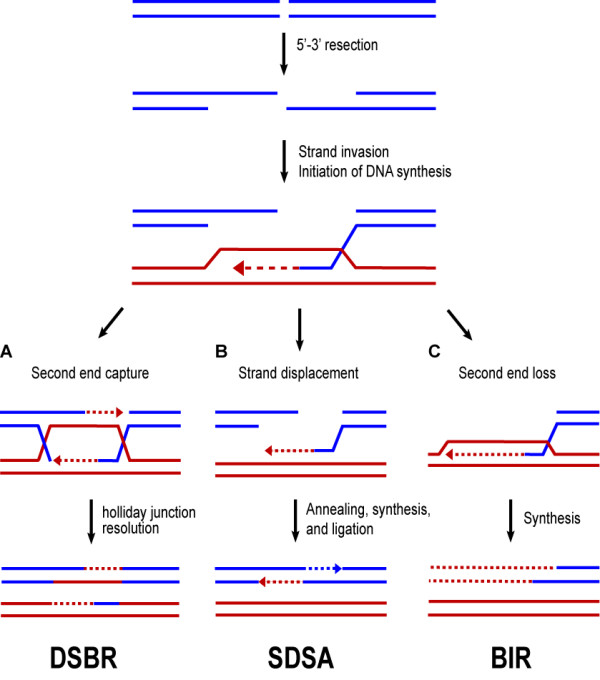
**Models of homologous recombination**. DSBs can be repaired using the homologous recombination machinery in a variety of ways. The DNA ends are first processed into 3' ssDNA tails. These tails invade a homologous template (red) priming new DNA synthesis (dashed line). Three possible outcomes from this invasion are shown. **A) **In canonical DSBR, both the initial invading strand and the captured second end anneal to the homologous template and prime new DNA synthesis, resulting in a double Holliday junction that can be resolved by nucleases into crossover or non-crossover products (non-crossover product shown). **B) **Alternatively, after the single ssDNA tail invades the homologous template, a round of DNA synthesis is primed from the 3' end (dashed red line). Synthesis-dependent strand annealing (SDSA) occurs when the invading strand, along with the newly synthesized segment, is unwound by a helicase and annealed with the other resected end. **C) **In break-induced replication (BIR), one end of the DSB is lost and the remaining end invades the homologous template priming DNA synthesis to the end of the chromosome.

Homologous recombination, on the other hand, rejoins the DNA ends faithfully using a homologous template for repair and requires the Rad52 epistasis group of proteins [4,5]. Initiation of HR begins with processing of the DSB ends by one or more nucleases into 3' single strand DNA (ssDNA) tails [4,15,16]. The ssDNA is bound by RPA, which also recruits the checkpoint complex Mec1-Ddc2, homologs of vertebrate ATR-ATRIP respectively [6,17]. Rad52, a central component of the yeast HR machinery, catalyzes the assembly of the RecA homologue Rad51 into a long nucleoprotein filament, the displacing RPA from the ssDNA tails [18-21]. This filament initiates the homology search using the sequence to be repaired--the "acceptor"--to find a repair template--the "donor" of genetic information. Once a homologous template is found, the nucleoprotein filament engages in strand invasion involving the Swi/Snf homolog Rad54 [22]. The Rad55/Rad57 complex stabilizes the Rad51/DNA filament promoting strand pairing and exchange [23,24]. Figure 2 shows three possible outcomes for the repair of a DSB by HR after invasion of one end. In canonical double-strand break repair (DSBR) (Figure 2A), second end capture--likely catalyzed by the annealing activity of Rad52--results in both ends of the DSB invading the acceptor [25]. DNA replication primed from both donor 3' ends leads to the formation of a joint molecule containing two Holliday junctions. In synthesis-dependent strand annealing (SDSA, Figure 2B) new DNA synthesis occurs along only one strand, which is subsequently displaced by a DNA helicase. The resultant ssDNA tail contains complementary sequence capable of annealing to the other DSB end. In this case, an additional role for Rad52 is to catalyze annealing to form duplex DNA between the 3' tails [26-28]. Any resulting gaps are filled by DNA replication. In the absence of second-end capture and/or SDSA, break-induced replication (BIR) can occur (Figure 2C). In BIR, the single DNA end that invades the homologous chromosome primes new DNA synthesis, which proceeds until it reaches the end of the chromosome [4,5,29-31].

## Cell cycle regulation of homologous recombination

Double-strand break repair is highly coordinated with the cell cycle: NHEJ occurs primarily in G1 while HR takes place predominantly during S and G2/M [3,4,7,32,33]. The major cell cycle kinase, CDK1--Cdc28 in *S. cerevisiae*--lies at the heart of the cell cycle regulation of DSBR CDK1 regulates the initiation of HR at two distinct levels: 1) DNA resection and 2) recruitment of Rad52 itself. Extensive resection of DSB ends only occurs in the presence of the high CDK1 activity in S and G2/M [34-36]. The MRX-associated nuclease Sae2--CtIP in vertebrates--aids in the processing of broken DNA ends along with the helicase Sgs1, and the Exo1 and Dna2 nucleases [37-42]. CDK1 phosphorylates Sae2, promoting highly processive DNA resection upon entry into S phase [43]. The result is long tracts of 3' ssDNA flanking the break site. Relocalization of repair proteins to DSBs, such as RPA, can be visualized as subnuclear foci [44]. When RPA binds ssDNA exposed in G1 cells, the foci formed are observably smaller and less intense than those in S and G2/M cells, perhaps reflecting the difference in resection rate between these cell cycle states [45].

During S and G2/M, Rad52 recruitment is dependent upon RPA, although RPA bound to ssDNA is not sufficient for Rad52 recruitment in G1 [6,45]. Studies have shown that CDK1 activity is also required for the recruitment of Rad52, even in the presence of RPA-bound ssDNA [46,47]. Interestingly, Rad52 recruitment to DSB sites does not rely on DNA replication *per se*, as cells that have entered S phase, but have not replicated their DNA, readily form Rad52 foci in response to DNA damage [46,47]. Exactly how CDK1 regulates this process is unknown. Perhaps CDK1 acts directly on the Rad52 protein itself or phosphorylates an upstream factor like RPA. Both of these proteins are good candidates since--like Rad52 (see below)--Rfa2 is also phosphorylated in a cell cycle-dependent manner and in response to genotoxic stress [48-53]. In addition, phosphorylation of the N-terminus of human Rpa2 by CDK1 is important for DNA repair as cells expressing a non-phosphorylatable form of Rpa2 exhibit an altered cell cycle profile and a persistent DNA damage signal [54].

## Regulation of HR during DNA replication

**Figure 3:**
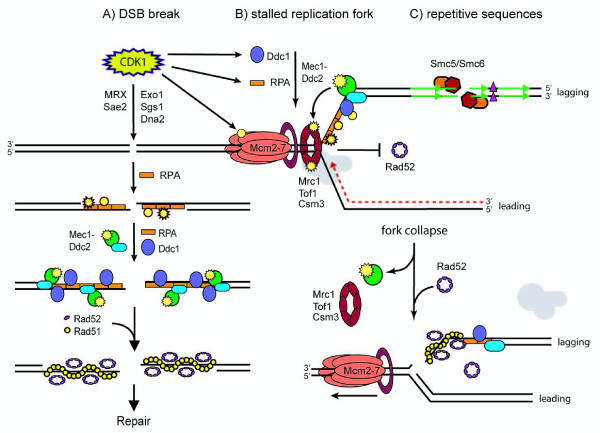
**Regulation of HR**. Recruitment of the HR machinery to a DSB is regulated by both the major cell cycle kinase CDK1 and the checkpoint kinase Mec1. CDK1 phosphorylation is marked as a yellow circle and Mec1 phosphorylation is marked as a yellow star. **A) **Once a DSB is detected, the DNA ends are resected forming 3' ssDNA tails by multiple nucleases that are positively regulated by CDK1/B type cyclin kinase activity. RPA binds the ssDNA and recruits the ATR-ATRIP homolog Mec1-Ddc2 and the 9-1-1 complex comprised of Ddc1, Mec3 and Rad17 (indicated by Ddc1). Finally, Rad52 catalyzes the formation of a Rad51 nucleoprotein filament along the ssDNA before HR can proceed. **B) **The intra-S phase checkpoint proteins Mrc1, Tof1 and Csm3 travel with the fork during normal replication. In response to DNA damage, the replication fork stalls, activating Mec1 which in turn phosphorylates Mrc1. Phosphorylated Mrc1 promotes stable fork pausing and contributes to Mec1 retention at the fork. In the absence of Mec1, the replisome is not stable when the fork pauses or stalls, leading to the uncoupling of the MCM helicase and the polymerase (grey) and fork collapse (bottom right). The DNA replication clamp PCNA is the circle adjacent to MCM2-7. Rad52 is recruited to the collapsed fork and HR restarts replication by one-end invasion of the intact DNA molecule (here shown as lagging strand invasion of the leading strand template). **C) **In repetitive sequences (indicated by green arrows) the Smc5/6 complex is recruited to DSBs along with the DSBR machinery, shown in A, to mediate repair. Smc5/6 and the sumoylation state of Rad52 affect whether repair deletes or retains DNA sequences between repeats (purple triangles) during direct repeat recombination.

Cells are most vulnerable to genomic insults during DNA replication and entry into S phase. In preparation for DNA replication, a series of protein complexes bind sequentially to specific DNA sequences in a process termed origin licensing [55-58]. Proper origin licensing during G1 is essential for rapid and faithful DNA replication during S phase, as origins are unable to re-license until the cell has progressed through the cell cycle to M phase [59]. DNA replication initiates by origin firing, when the replisome--including the DNA helicase encoded by Mcm2-7, the DNA replication clamp PCNA and both the leading and lagging strand polymerases--moves away from origins and DNA synthesis begins. Damage can arise during replication by mechanical stress on the DNA itself or if the replisome encounters covalently attached DNA adducts. Lesions may also arise if replication does not proceed efficiently, leading to the uncoupling of DNA unwinding and new DNA synthesis. To maintain genomic integrity, a number of mechanisms have evolved both to stabilize DNA during replication and to activate specific S phase checkpoints in the event of DNA damage (see Figure 3).

The checkpoint kinase Mec1 and its activation partner Ddc2 play key roles in genome stability during S phase. Mec1 kinase activity and its downstream effector kinase Rad53 are required to stabilize stalled forks and activate the intra-S phase checkpoint [60-62]. Mec1 phosphorylates Mrc1, an intra-S phase checkpoint protein associated with the replication fork, and promotes restart of stalled forks [63-65]. Furthermore, Mrc1 is required for the accumulation of Mec1 at stalled forks, independently of Rad53 activity [63]. Mrc1 physically inhibits the uncoupling of the replicative helicase from the leading strand polymerase, locking both halves of the replisome at the stalled fork [53,66]. How these checkpoint complexes interact with the recombination machinery in S phase is not well understood.

Hydroxyurea (HU), a potent ribonucleotide reductase inhibitor, decreases dNTPs pools and stalls replication forks, activating the intra-S phase checkpoint. In the presence of HU, Rad52 is unable to form either spontaneous or damage-induced recombination foci [6,46,47]. In addition, the rate of resection at DSBs is significantly reduced during HU-mediated S phase arrest, leading to a reduction in ssDNA formation [46]. One argument is that the amount of RPA-bound ssDNA is limited and may not be sufficient to recruit Rad52. However, even during HU arrest, IR-induced DNA breaks undergo enough resection to form RPA foci and still do not form Rad52 foci [47]. These results suggest that a simple model hinging on available RPA-bound ssDNA does not fully explain the complete absence of Rad52 foci Therefore we propose that the intra-S phase checkpoint imposes an additional level of regulation, suppressing Rad52 foci both at stalled replication forks and at DSBs. This regulation is likely performed by Mec1, since suppression of Rad52 foci in HU is Mec1/Rad53-dependent [47]. However Rad52 is not a direct target of either the Mec1 or Rad53 kinases ([67]; AAM, unpublished data). Interestingly, in *S. pombe*, Rad52 does associate with stalled replication forks; however, the levels are too low to form observable foci [68]. These results indicate that low-level Rad52 association is important to initiate recombination in case of fork collapse, although the precise function of Rad52 at stalled forks is unknown. In budding yeast, it is clear that the recruitment of the HR machinery to stalled forks is detrimental to cell survival. This notion is supported by the fact that HR is toxic in the absence of proteins necessary to resume replication, in particular Top3, Sgs1, and Srs2 [69-72].

Although HR is inhibited at stalled replication forks, it is necessary to permit HR during S phase for cell survival in the event of fork collapse. In support of this idea, spontaneous Rad52 foci are observed in ~50% of S phases [73]. The HR machinery--in particular Rad51 and Rad52--plays an important role in replication fork restart, an event where a one-ended DNA fragment is generated (Figure 3; [4,5]). Interestingly, this view of replication fork restart resembles BIR, which also couples Rad52-mediated invasion of a one-ended DNA break with extensive DNA replication (Figure 2C). Furthermore, loss of either Rad51 or Rad52 is synthetic lethal In a Mec1- or Mrc1-deficient background, suggesting that both of these proteins are required for the restart of collapsed forks [74,75]. Thus, repair of the resulting DNA break requires many of the same components involved in canonical DSBR (Figure 3).

## Regulation of Rad52 activity by posttranslational modification

The *S. cerevisiae *Rad52 protein is phosphorylated both constitutively and upon entry into S phase. These phosphorylation events occur in the C terminus, although the exact residues and phenotypic effects of loss of phosphorylation have yet to be determined [48]. Interestingly, Rad52 does not exhibit further phosphorylation upon exposure to DNA damaging agents. Furthermore, these multiple phosphorylation events are Mec1 and Tel1 independent, underscoring their damage-independent nature [48]. Although CDK1 activity is required for Rad52 recruitment to foci, it is unclear whether CDK1 is directly responsible for Rad52 cell cycle-dependent phosphorylation. A tantalizing possibility is that CDK1-dependent phosphorylation of RPA regulates Rad52 recruitment only after the cell has entered into S phase.

In addition to phosphorylation, Rad52 also undergoes sumoylation on its N terminus and in the central domain at lysines 10, 11 and 220, in an Ubc9-dependent manner [76]. Mutation of these residues leads to a loss of sumoylation on Rad52, yet it does not affect overall HR levels or Rad52 recruitment to DNA damage. On the other hand, loss of Rad52 SUMO species does decrease protein stability and affect the outcome of direct repeat recombination, specifically decreasing events that delete intervening DNA [76]. In the absence of Rad52 sumoylation, recombination at rDNA is also affected, a locus consisting of 100-200 tandem repeats of the ribosomal genes that resides in the nucleolus. Non-sumoylatable Rad52 forms foci in the nucleolus and shows an increase in rDNA recombination, where wild-type Rad52 foci are normally excluded from the nucleolus [77]. Interestingly, regulation of Rad52 focus formation at rDNA also requires the Smc5/6 complex, which removes cohesion during mitotic exit for proper chromosomes segregation and is itself involved in sumoylation [78,79]. Together, these results suggest a link between the resolution of recombination intermediates at highly repetitive DNA to chromosome segregation during mitosis.

## Conclusions

Regulation of Rad52 affects double strand break repair by initiating and/or directing many aspects of HR. Cell cycle regulation of its recruitment inhibits initiation of homologous recombination processes in G1. Checkpoint regulation of Rad52 blocks its recruitment to stalled DNA replication forks allowing them to restart independently of HR. These regulatory mechanisms help suppress potentially mutagenic or lethal recombination events in different ways. Limiting resection of the DNA ends and inhibiting the recruitment of HR proteins in G1 allows cells to repair DSBs by NHEJ with little to no information lost at the break site. After DNA replication however, the cell contains a sister chromatid, which is an identical template for repair and thus recombination is non-mutagenic (Figure 1). In diploid cells, repair can proceed from the homologous chromosome potentially leading to loss of heterozygosity that impacts the oncogenic transformation of cells by homozygosing deleterious mutations, resulting in widespread genomic instability.

However, the cell does not wait for the completion of replication to begin HR, as Rad52 is recruited into damage-induced foci early during S phase [47,73]. Entry into S phase may present a point in the cell cycle where the repair of a potentially lethal lesion such as a DSB that occurred in G1 must undergo non-conservative recombination before replication can proceed. At this stage, the cell may repair the DSB using the homologous chromosome as a template, again with the potential for loss of heterozygosity. Alternatively, the break can be repaired using a non-homologous or homeologous sequence to promote cell survival. Depending on the choice of the sequence used to template the repair, the outcome may result in the loss of DNA flanking the break site. Furthermore, SUMO-modified forms of Rad52 affect DNA repair within repetitive sequences. How these alternate forms of Rad52 mediate template choice is unclear and elucidating their roles will impact our understanding on how DNA repair proceeds.

Finally, it is not only necessary to restrict the activity of recombination to different stages of the cell cycle, it is also important to complete the process appropriately. Future studies will clarify how Rad52 and other members of the HR machinery dissociate from the repaired DNA, adding another level of Rad52 regulation. For example, the Srs2 helicase antagonizes Rad51 filament formation that occurs independently of Rad52 but does not efficiently remove Rad52 protein, which may mark true recombination sites [80]. Other chromatin remodeling enzymes/complexes such as Rdh54, Ino80 or Rsc may also be involved in the dissolution of HR complexes. In the end, the co-ordination of recombination in budding yeast revolves around the Rad52 protein for integration of checkpoint and cell cycle signals necessary to coordinate an appropriate repair response.

## Competing interests

The authors declare that they have no competing interests.

## Authors' contributions

JHB and RR drafted and approved the final manuscript.
